# Progress Toward Rubella and Congenital Rubella Syndrome Control and Elimination — Worldwide, 2000–2018

**DOI:** 10.15585/mmwr.mm6839a5

**Published:** 2019-10-04

**Authors:** Gavin B. Grant, Shalini Desai, Laure Dumolard, Katrina Kretsinger, Susan E. Reef

**Affiliations:** ^1^Global Immunization Division, Center for Global Health, CDC; ^2^Department of Immunization, Vaccines and Biologicals, World Health Organization, Geneva, Switzerland.

Rubella is a leading cause of vaccine-preventable birth defects. Although rubella virus infection usually causes a mild febrile rash illness in children and adults, infection during pregnancy, especially during the first trimester, can result in miscarriage, fetal death, stillbirth, or a constellation of birth defects known as congenital rubella syndrome (CRS). A single dose of rubella-containing vaccine (RCV) can provide lifelong protection (*1*). In 2011, the World Health Organization (WHO) updated guidance on the use of RCV and recommended capitalizing on the accelerated measles elimination activities as an opportunity to introduce RCV (*1*). The Global Vaccine Action Plan 2011–2020 (GVAP) includes a target to achieve elimination of rubella in at least five of the six WHO regions by 2020 (*2*). This report on the progress toward rubella and CRS control and elimination updates the 2017 report (*3*), summarizing global progress toward the control and elimination of rubella and CRS from 2000 (the initiation of accelerated measles control activities) and 2012 (the initiation of accelerated rubella control activities) to 2018 (the most recent data) using WHO immunization and surveillance data. Among WHO Member States,* the number with RCV in their immunization schedules has increased from 99 (52% of 191) in 2000 to 168 (87% of 194) in 2018^†^; 69% of the world’s infants were vaccinated against rubella in 2018. Rubella elimination has been verified in 81 (42%) countries. To make further progress to control and eliminate rubella, and to reduce the equity gap, introduction of RCV in all countries is important. Likewise, countries that have introduced RCV can achieve and maintain elimination with high vaccination coverage and surveillance for rubella and CRS. The two WHO regions that have not established an elimination goal (African [AFR] and Eastern Mediterranean [EMR]) should consider establishing a goal.^§^

## Immunization Activities

The preferred strategy for introducing RCV into national immunization schedules is to conduct an initial vaccination campaign targeting the majority of persons who might not have been naturally exposed to rubella, usually children aged ≤14 years (*1*), a strategy that can eliminate rubella and CRS (*4*). WHO recommends that countries that introduce RCV achieve and maintain a minimum coverage of at least 80% with at least 1 dose of RCV, delivered through routine services or campaigns (*1*). Financial resources to introduce RCV are provided by governments, and Gavi, the Vaccine Alliance (Gavi) also provides substantial support for low-income and some lower-middle–income countries.

Each year, countries report immunization data to WHO and the United Nations Children’s Fund using the Joint Reporting Form, which includes information on immunization schedules and the number of vaccine doses administered through routine immunization services and vaccination campaigns.^¶^ RCV was available in high-income countries before becoming available in lower-income countries. World Bank country income groupings were used to assess RCV introduction among countries in different income categories.**

**FIGURE 1:**
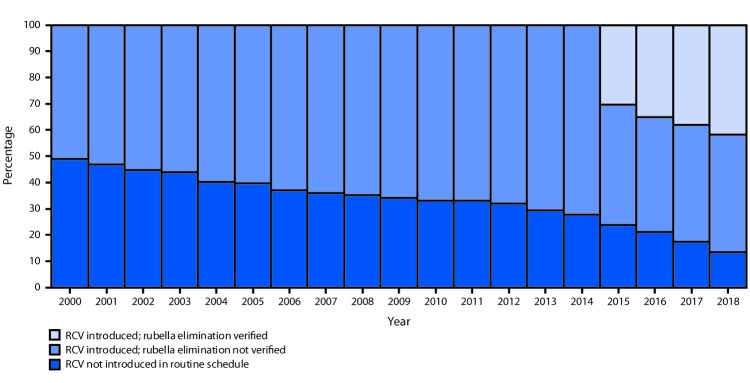
Percentage of countries that have introduced rubella-containing vaccine (RCV) and the percentage with verified rubella elimination, by year — worldwide, 2000–2018

According to Joint Reporting Form data, global coverage of infants with RCV increased from 21% in 2000 to 40% in 2012 and to 69% in 2018 (Table). In 2000, approximately half (52%, 99 of 191) of countries had introduced RCV into national immunization schedules. By the end of 2012, approximately two thirds (68%, 132 of 194) of countries were using RCV and by 2018, 168 (87%) countries had introduced RCV (Figure 1). WHO recommends that RCV be given with the first routine dose of measles-containing vaccine (MCV1) (i.e., as a combination vaccine). This recommendation has been implemented in 163 (97%) of the 168 countries that have introduced RCV; one country introduced the vaccine before the recommendations were published, and four countries administer monovalent measles vaccine at age 9 months and RCV as a combination measles-mumps-rubella vaccine at age 12 months, which is consistent with licensed use.

**TABLE:** Global progress toward control and elimination of rubella and congenital rubella syndrome (CRS) by World Health Organization (WHO) regions — worldwide, 2000, 2012 and 2018

Characteristic	WHO region (no. of countries)						
	AFR (47)	AMR (35)	EMR (21)	EUR (53)	SEAR (11)	WPR (27)	Worldwide (194)*
Regional rubella/CRS target	None	Elimination	None	Elimination	Control	Elimination	None
No. (%) of countries verified eliminated*							
2000	N/A	N/A	N/A	N/A	N/A	N/A	N/A
2012	N/A	N/A	N/A	N/A	N/A	N/A	N/A
2018	N/A	35 (100)	3 (14)	39 (74)	6^†^ (55)	4 (15)	81 (42)
**No. (%) of countries with RCV in schedule**							
2000	2 (4)	31 (89)	12 (63)	40 (77)	2 (20)	12 (44)	99 (52)
2012	3 (6)	35 (100)	14 (67)	53 (100)	5(45)	22 (81)	132 (68)
2018	27 (57)	35 (100)	16 (76)	53 (100)	10 (91)	27 (100)	168 (87)
**Regional rubella vaccination coverage (%)^§^**							
2000	0	85	23	60	3	11	21
2012	0	94	38	95	5	86	40
2018	32	90	45	95	83	94	69
**No. (%) of countries reporting rubella cases**							
2000	7 (15)	25 (71)	11 (52)	41 (79)	3 (30)	15 (56)	102 (53)
2012	41 (87)	35 (100)	19 (90)	47 (89)	11 (100)	23 (85)	176 (91)
2018	45 (96)	34 (97)	18 (86)	46 (87)	11 (100)	22 (81)	176 (91)
**No. of reported rubella cases**							
2000	865	39,228	3,122	621,039	1,165	5,475	670,894
2012	10,850	15	1,681	30,579	6,877	44,275	94,277
2018	11,787	2	1,622	798	4,533	7,264	26,006
**No. (%) of countries reporting CRS cases**							
2000	3 (7)	18 (51)	6 (29)	34 (65)	2 (20)	12 (44)	75 (39)
2012	20 (43)	35(100)	9 (43)	43 (81)	6 (55)	17 (63)	130 (67)
2018	19 (40)	33 (94)	13 (62)	46 (87)	10 (91)	17 (63)	138 (71)
**No. of reported CRS cases**							
2000	0	80	0	47	26	3	156
2012	69	3	20	62	14	134	302
2018	18	0	39	14	342	36	449

**Abbreviations:** AFR = African Region; AMR = Region of the Americas; EMR = Eastern Mediterranean Region; EUR = European Region; N/A = not available; RCV = rubella-containing vaccine; SEAR = South-East Asia Region; WPR = Western Pacific Region.

* In 2000, WHO had 191 Member States worldwide; one country was added in each of three regions (AFR, EUR, and SEAR) by 2012, resulting in 194 countries.

^†^ Established regional verification commissions verify achievement of elimination in four regions (AMR, EMR, EUR, and WPR), but verify control in one (SEAR). The six countries in SEAR that have been verified as controlled are not included in the worldwide total of countries eliminated.

**^§^** Coverage estimates for rubella-containing vaccines are determined by WHO and United Nations Children’s Fund Estimate National Immunization Coverage.

All countries in the Region of the Americas (AMR), the Western Pacific Region (WPR) and the European Region (EUR) have introduced RCV. In the remaining regions, RCV has been introduced in 27 (57%) of 47 countries in AFR, 16 (76%) of 21 countries in EMR, and 10 (91%) of 11 countries in the South-East Asia Region (SEAR) (Table).

**FIGURE 2:**
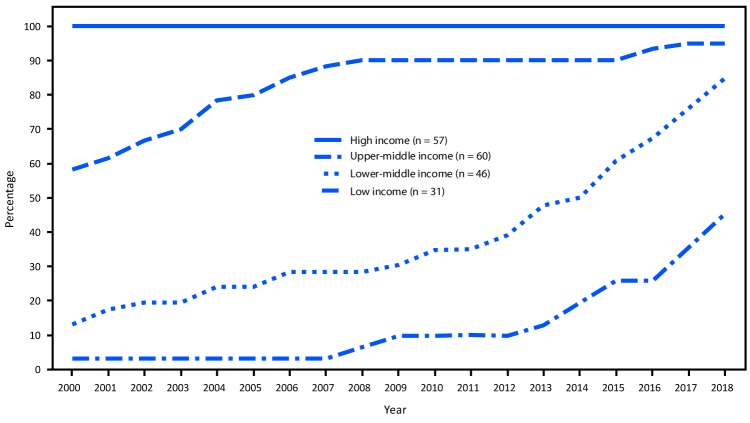
Percentage of countries that have introduced rubella-containing vaccine, by World Bank income group* and year — worldwide, 2000–2018 * Gross National Income per capita in USD in 2018: high income: >$12,055; upper-middle income: $3,896–12,055; lower-middle income: $996–$3,895; low income: <$995). https://blogs.worldbank.org/opendata/new-country-classifications-income-level-2019-2020.

The income group of countries introducing RCV has shifted over time (Figure 2). In 2000, RCV had been introduced in all 57 high-income countries but in only 13% of lower-middle–income countries and 3% of low-income countries. By 2018, 39 (85% of 46) lower-middle–income countries and 14 (45% of 31) low-income countries had introduced RCV. Fifteen countries introduced RCV in 2017 and 2018, including 14 that used financial support from Gavi (Supplementary Table, https://stacks.cdc.gov/view/cdc/81634).

## Surveillance Activities

Surveillance data for rubella and CRS are also reported through the Joint Reporting Form using standard case definitions (*5*). Rubella and CRS surveillance data complement each other to provide a better picture of program progress. Rubella surveillance uses the measles surveillance system to detect cases because both illnesses cause fever and rash; however, rubella is typically milder than measles, resulting in a lower proportion of persons infected with rubella seeking health care for the illness, and therefore being detected. CRS cases are detected through separate surveillance systems, often using a few sentinel sites, which are not nationally representative (*6*).

The number of countries reporting rubella case counts, including reports of zero cases, increased from 102 (53%) in 2000 to 176 (91%) in 2012 and 2018 (Table). The number of countries reporting CRS case counts has also increased from 75 (39%) in 2000 to 130 (67%) in 2012 and to 138 (71%) in 2018. Compared with the 670,894 rubella cases reported in 2000, case counts declined by 86% in 2012 and by 96% in 2018.

## Progress Toward Elimination

Progress toward regional goals is measured by the number of countries introducing RCV and the number verified as having eliminated rubella and CRS. Rubella elimination is defined as the interruption of endemic rubella virus transmission for at least 12 months. When interruption of transmission is sustained for 36 months, an independent regional commission verifies countries as having eliminated rubella (*7*). Data on verification of elimination are from regional verification commission reports^††^^,^^§§^ (*8*,*9*).

Rubella and CRS regional elimination goals have been established by AMR, EUR, and WPR; a control goal has been established by SEAR^¶¶^; AFR and EMR do not yet have a goal. The AMR commission verified the entire AMR to have eliminated rubella and CRS in 2015; verification commissions in EMR, EUR, and WPR assess rubella elimination status country-by-country. The elimination of endemic rubella has been verified in 81 countries: three of 23 (13%) in EMR, 39 of 53 (74%) in EUR, four of 27 (15%) in WPR, and 35 (100%) in AMR. In SEAR, six of 11 (55%) countries were verified to have achieved the regional control goal of a 95% reduction in cases.

## Discussion

Progress toward rubella elimination has accelerated since 2011 with the establishment of new WHO rubella elimination goals and the availability of Gavi financial support for RCV introduction. Progress is reflected by an increase in the number of countries introducing RCV into national childhood immunization schedules and the coverage achieved, from 99 countries in 2000 (21% global RCV coverage) to 132 in 2012 (40% coverage) and 168 in 2018 (69% coverage). The equity gap in access to RCV among countries has narrowed as more middle-income and low-income countries have introduced RCV, in part with funding from Gavi to support activities required for introduction; however, inequities remain among countries and at subnational levels.

Providing policy-makers in countries that have not yet introduced RCV with data on the impact of the investment to introduce RCV can help them determine whether their country should introduce RCV. The decision-making process benefits from 1) evaluation of the impact of RCV introduction on CRS; 2) consideration of the opportunities offered by accelerated measles elimination activities (e.g., campaigns); and 3) evaluation of the long-term sustainability of financing for RCV along with other vaccines. It is important that all countries that have not reached >95% measles-containing vaccine coverage (the level needed to achieve measles elimination) continue to improve population immunity with high-coverage routine services and campaigns and, by doing so, also eliminate rubella. In addition, countries that had introduced RCV in selected populations (usually females only) to control CRS, have large immunity gaps (usually in men) and might need to develop plans to identify and protect susceptible populations to achieve elimination. Research and innovation will help improve surveillance, target programmatic activities more effectively, and develop new vaccination delivery systems to help further accelerate progress toward rubella and measles elimination (*10*).

The findings in this report are subject to at least two limitations. First, improvements in the accuracy and reliability of available surveillance and immunization data are needed to better identify immunity gaps, to focus immunization-strengthening activities, and to demonstrate the interruption of rubella virus transmission. Second, the impact of recent RCV introductions (e.g., two large countries in SEAR introducing RCV in 2018) might not be fully reflected in the available surveillance data.

Increases in the number of countries introducing RCV into national immunization schedules, in global RCV coverage, and in the number of countries verified as having eliminated endemic rubella transmission demonstrate the progress toward control and ultimately the elimination of rubella. The countries verified as having eliminated rubella serve as important examples and provide valuable lessons for other countries. Countries in all income groups can eliminate rubella by introducing RCV, strengthening surveillance, and improving immunization service delivery.

**Summary**

**What is already known about this topic?**

Congenital rubella syndrome is caused by rubella virus infection of pregnant women. Since 2011, there has been an acceleration in the efforts to introduce rubella-containing vaccine using a strategy that can result in elimination.

**What is added by this report?**

Progress toward rubella elimination has resulted in 168 (87%) of 194 countries protecting infants with RCV and 81 (42%) eliminating rubella transmission. Equity between countries using rubella-containing vaccine has increased as lower-income countries have introduced rubella-containing vaccine.

**What are the implications for public health practice?**

To make further progress, it is important that the 26 remaining countries introduce rubella vaccine and the countries that have already introduced the vaccine achieve and maintain elimination.

## Footnotes

* In 2000, WHO had 191 Member States worldwide; one country was added in each of three regions (African Region, European Region, and South-East Asia Region) by 2012, resulting in 194 Member States.

† One country (Indonesia) is categorized as having introduced RCV in 2018 although introduction began in 2017 and was completed by the end of 2018.

§ In 2019, the South-East Asia Region established an elimination goal, leaving two regions without an elimination goal at the time of publication.

¶ https://www.who.int/immunization/monitoring_surveillance/routine/reporting/en/.

** World Bank Gross National Income classification cut-offs per capita in USD in 2018: high income >$12,055; upper-middle income = $3,896–$12,055; lower-middle income = $996–$3,895; low income ≤$995). https://blogs.worldbank.org/opendata/new-country-classifications-income-level-2019-2020.

†† http://www.emro.who.int/media/news/rvc-declared-bahrain-oman-iran-rubella-measles-free.html.

§§ http://www.euro.who.int/__data/assets/pdf_file/0009/410967/8th-RVC-report-annex.pdf.

¶¶ Rubella and CRS control is defined as a 95% reduction of rubella and CRS cases, compared with the 2010 baseline nationally and regionally.
